# Spirulina Preconditioning Attenuates Ischemia–Reperfusion Injury in a Steatotic Rat Liver Model

**DOI:** 10.3390/antiox15030390

**Published:** 2026-03-19

**Authors:** Eya Baily, Kamel Mhalhel, Soumaya Ben Ahmed, Mohamed Amine Zaouali, Giuseppe Montalbano, Ines Naouar, Antonino Germanà, Hassen Ben Abdennebi

**Affiliations:** 1 Laboratory of Human Genome and Multifactorial Diseases (LR12ES07), Faculty of Pharmacy of Monastir, Higher Institute of Biotechnology of Monastir, University of Monastir, Monastir 5000, Tunisia; aya.baily@fphm.u-monastir.tn (E.B.); soumaya.benahmed@isbm.u-monastir.tn (S.B.A.); mohamedamine.zaouali@isbm.u-monastir.tn (M.A.Z.); ines.naouar@fphm.u-monastir.tn (I.N.); hassen.benabdennebi@fphm.u-monastir.tn (H.B.A.); 2 Zebrafish Neuromorphology Laboratory, Department of Veterinary Sciences, University of Messina, Polo Universitario dell’ Annunziata, 98168 Messina, Italy; agermana@unime.it

**Keywords:** spirulina, steatosis, inflammation, liver dysfunction, ischemia, reperfusion, pyroptosis, histology, oxidative stress

## Abstract

Ischemia and reperfusion (IR) injuries may produce deleterious effects on hepatic tissue after liver surgery and transplantation. The consequences of IR are more evident in pathological steatotic livers. Spirulina (*Arthrospira platensis*) is known for its potential to modulate inflammatory responses and enhance antioxidant defenses. The current investigation assessed whether spirulina pretreatment mitigates hepatic IR injury exacerbated by steatosis in rats. Thirty male Wistar rats were divided into five groups: sham, IR, HFD, HFD + IR, and SP1000 (HFD + IR + spirulina 1000 mg/kg/day; oral gavage). Liver injury, oxidative stress, inflammatory signaling, and inflammasome/pyroptosis-related markers were assessed using serum transaminases, hematoxylin–eosin staining, immunofluorescence, and qRT-PCR. High-fat diet-fed rats developed steatosis, which significantly worsened IR-induced liver damage, as shown by the respective steatosis histological score, the elevated alanine aminotransferase (ALT) and aspartate aminotransferase (AST), and higher expression of inflammatory markers, including Toll-like receptor (*TLR4*), nuclear factor kappa B (*NF-κB*), tumor necrosis factor alpha (*TNF-α*), and interleukin-1 beta (*IL-1β*) and inflammasome/pyroptosis-related transcripts, namely NOD-like receptor family pyrin domain-containing 3 (*NLRP3*), interleukin-18 (*IL18*), and gasdermin D (*GSDMD*). Oxidative stress was exacerbated, as reflected by higher levels of malondialdehyde (MDA) and reduced antioxidant defenses (superoxide dismutase (SOD) activity, reduced glutathione (GSH) content, glutathione peroxidase (*GPx*) expression, and heme oxygenase-1 (*HO-1*) expression). Furthermore, HFD + IR upregulated sterol regulatory element-binding protein-1c (*SREBP-1c*) expression and downregulated AMP-activated protein kinase (*AMPK*) expression. Spirulina supplementation significantly attenuated liver injury and transaminase release, reduced MDA, restored antioxidant parameters, downregulated inflammatory and inflammasome-related gene expression, and shifted both *SREBP-1c* and *AMPK* expressions toward control levels.

## 1. Introduction

Non-alcoholic fatty liver disease (NAFLD) has become one of the most prevalent chronic liver disorders worldwide [1]. Its global prevalence in adults has risen over time, increasing from approximately 26% in studies conducted up to 2005 to 32% in studies published from 2016 [2,3]. The pathological features of NAFLD impair hepatic microcirculation, prime Kupffer cells, and limit the liver’s regenerative capacity, thereby rendering steatotic livers particularly more vulnerable to the oxidative and inflammatory cascades [4,5]. Similar alterations have also been reported in high-fat diet (HFD)-fed rat models of hepatic steatosis and metabolic dysfunction. Although species-specific differences in metabolism, immune regulation, and disease progression exist, the HFD-induced steatosis model provides a mechanistically informative and translationally relevant platform for investigating metabolic–inflammatory interactions and evaluating hepatoprotective strategies in the context of human liver disease [6].

As NAFLD progresses to advanced liver disease in a growing subset of patients, its impact on liver transplantation is expanding, both by increasing recipient demand and by reshaping the donor pool [7]. Hepatic ischemia–reperfusion (IR) injury is a frequent and clinically significant complication during liver resection, transplantation, and trauma surgery. Damage induced by IR elevates the risk of early allograft dysfunction and rejection, accounting for around 10% of early transplant failures [8]. Mechanistically, hepatic IR injury is driven by mitochondrial dysfunction and a burst of reactive oxygen species (ROS), leading to lipid peroxidation and depletion/inactivation of antioxidant defenses, amplifying sterile inflammation by engaging innate immune signaling pathways, including the *TLR4*/*NF-κB* signaling cascade [9]. More recently, inflammasome activation has been proven to be implicated in steatotic IR injury. Experimental evidence indicates that multiple regulated cell-death pathways operate concurrently, and pyroptosis signaling, particularly in immune cells, amplifies the inflammatory response [10]. A major challenge is that donor livers with macrovacuolar steatosis are increasingly being considered to alleviate the shortage of transplants, despite the fact that steatosis significantly reduces the liver’s tolerance to IR injury [9]. Despite advances in preservation and perioperative care, few practical interventions are available to specifically protect steatotic livers against IR injury [11]. Therefore, the development of strategies for alleviating IR injury in steatotic donor livers is of great importance for expanding the donor pool and improving transplantation outcomes. Natural compounds are widely recognized for their diverse pharmacological properties in limiting hepatic IR. Their hepatoprotective activities are attributed to their bioactive antioxidant constituents, which play a crucial role in mitigating oxidative stress and sterile inflammation [12]. Among these natural compounds, spirulina (*Arthrospira platensis*), a nutrient-dense cyanobacterium widely investigated for antioxidant and anti-inflammatory properties, is accredited to bioactive components such as phycobiliproteins (notably phycocyanin), carotenoids, phenolics, and micronutrients [4,13]. Accordingly, spirulina-based approaches warrant investigation in the context of hepatic ischemia. Building upon these findings, the present study aimed to investigate the potential hepatoprotective effects of spirulina preconditioning in a model of 70% hepatic IR injury in rats with diet-induced steatotic livers. In the current study, the potential effects of spirulina on hepatocellular injury, histological alterations, oxidative stress status, inflammatory signaling, inflammasome/pyroptosis-related gene expression, and transcriptional shifts in lipid metabolism have been assessed.

## 2. Materials and Methods

### 2.1. Animals

Thirty male Wistar rats of the same genetic background, aged 6–8 weeks old, and weighing 160–200 g, were purchased from the Tunisian Company for Pharmaceutical Industries (SIPHAT) (Ben Arous, Tunisia) and housed in the animal facility unit of the Faculty of Pharmacy of Monastir (University of Monastir, Tunisia) under controlled conditions (22 ± 2 °C and 12 h light/12 h dark cycle) with free access to water and food. The experiment was conducted respecting the Directive 2010/63/EU on the protection of animals used for scientific purposes and was approved by the “Research Ethics Committee for Life and Health Sciences of the Higher Institute of Biotechnology of Monastir” (approval reference: CER-SVS/ISBM 012/2024, date: 15 February 2025).

The study protocol including the research question, experimental design, and statistical analysis plan was prepared prior to the study initiation but was not registered in a public repository.

### 2.2. Experimental Design

**Figure 1:**
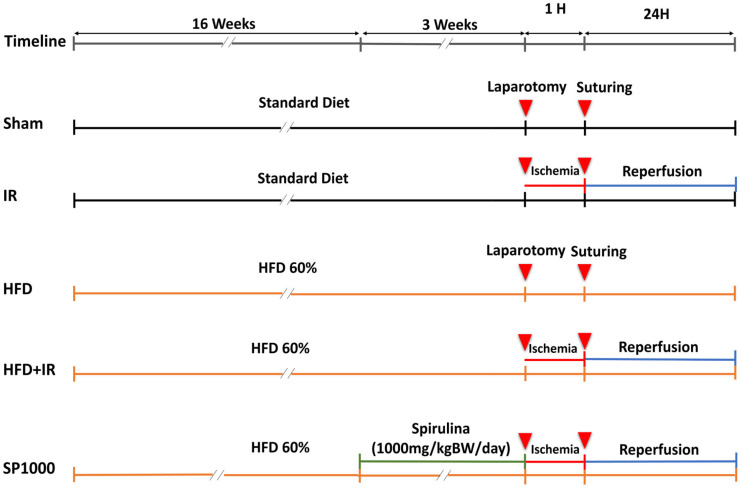
Experimental design. This figure illustrates the study timeline and treatment protocols for the five experimental groups: sham, IR (ischemia–reperfusion), HFD (high-fat diet), HFD + IR, and SP1000 (HFD rats subjected to IR and treated with 1000 mg/kg/day of spirulina). The procedure began with 16 weeks of dietary intervention (standard diet or 60%-fat diet), followed by a 3-week period where the SP1000 group received spirulina via oral gavage. The study concluded with a surgical phase consisting of 1 h of partial hepatic ischemia (or laparotomy only for the sham and HFD groups), followed by 24 h of reperfusion.

The 30 rats were randomly assigned using a computer-generated random number sequence to either a standard diet (Sd) (*n* = 12) or a high-fat diet (60% fat, *n* = 18) for 16 weeks to induce metabolic dysfunction and hepatic steatosis [14]. All experimental groups received the same feed amount (20 g/day at T0), adjusted to body weight gain as the rats were growing. The energy requirements were calculated according to Nutrient Requirements of Laboratory Animals [15]. Rats consistently consumed the full amount of feed provided; therefore, the energy intake was considered equivalent within each dietary group (Table S1). At the end of the feeding period (16 weeks), metabolic assessment (body weight gain and insulin resistance) was performed in a random order. Subsequently, standard-diet rats were randomly subdivided into sham and IR groups (*n* = 6 per group). High-fat diet rats were also randomly subdivided into HFD, HFD + IR, and SP1000 (HFD + spirulina + IR) groups (*n* = 6 per group). The animals continued their respective diets for an additional three weeks. During this period, the SP1000 group received spirulina (Bio Algues Tunisie, Ksour Essef, Mahdia, Tunisia) at a dose of 1000 mg/kg body weight/day through oral gavage in random order and at the same time each day [16]. At the end of the treatment period, the sham and HFD animals underwent laparotomy without vascular occlusion, whereas the IR, HFD + IR, and SP1000 groups underwent partial hepatic ischemia (70% of liver mass) for 60 min of ischemia, followed by 24 h of reperfusion, as summarized in Figure 1. The animals were monitored every 3 h following the surgery. Humane endpoints included uncontrolled bleeding, signs of severe pain or abdominal distension, and lack of recovery from anesthesia within the expected timeframe. A predefined clinical scoring sheet was implemented, and animals reaching a clinical score ≥ 10 were scheduled for humane euthanasia. However, no severe cases were recorded and no animal was excluded.

The investigators were blinded to group allocation during treatment administration, surgical procedures, and outcome assessment. Indeed, group assignments were concealed using coded identifiers that were not revealed until the completion of the data analysis.

#### Sample Size Determination

Sample size was determined a priori using serum glucose concentration as the primary outcome. Based on the published data, serum glucose levels were reported as 10.1 ± 1.01 mmol/L in the HFD group and 8.2 ± 0.45 mmol/L in the HFD + spirulina group [17]. Using these values, the pooled standard deviation was estimated at 0.78 mmol/L, corresponding to a mean difference of 1.9 mmol/L between groups.

The study was designed to detect this difference using the one-way ANOVA followed by Tukey’s post hoc multiple comparisons test, with a two-sided significance level of 0.05 and 95% statistical power. Thus, a sample size calculation using G*Power (version 3.1.9.7) indicated that a minimum of 6 animals per group was required.

### 2.3. Experimental Diets

The commercial standard diet was purchased from EL-BADR (Utique, Bizerte, Tunisia) and was checked for meeting the nutritional requirements of rats [15]. The diet provided approximately 12.1% of calories from fat, 26.3% from protein, and 61.1% from carbohydrates, with an energy density of 3340 kcal/kg. The high-fat diet consisted of a formulation in which animal fat derived from lamb accounted for approximately 60% of calories, and the remaining 11.3% and 31.939% of calories were derived from protein and carbohydrates, respectively (energy density of 4941 kcal/kg). Detailed macronutrient composition is provided in Table S1 (Supplementary Materials).

### 2.4. Metabolic Characterization

#### 2.4.1. Body Weight Gain

Body weight was recorded at baseline (T0) and after 16 weeks (T16) of the dietary intervention, prior to ischemia–reperfusion induction, using a calibrated digital balance (Adventurer™ Pro Precision, AV4101C; OHAUS Corporation, Parsippany, NJ, USA). The measurements were performed at the same time of day to minimize circadian variability.

#### 2.4.2. Insulin Tolerance Test (ITT)

Following 16 weeks of dietary intervention, the animals were fasted for 5 h prior to the metabolic assessment. Insulin sensitivity was evaluated through intraperitoneal injection of insulin (0.5 IU/kg body weight) dissolved in the saline solution. Blood glucose concentrations were measured from tail vein blood samples at 0 min (baseline, before insulin injection) and at 10, 20, 30, 45, and 60 min post-insulin injection using a calibrated glucometer Accu-Chek Active (Roche Diabetes Care, Hoffmann, Basel, Switzerland).

### 2.5. Hepatic Warm Ischemia–Reperfusion Model and Outcome Assessments

#### 2.5.1. Hepatic Warm Ischemia–Reperfusion Injury Model: Surgical Procedure

Throughout the surgical procedures, the rats were anesthetized with inhaled isoflurane (4% for induction, 1.5% for maintenance) using small-animal isoflurane vaporizer AD-5000 Digital Anesthesia System (E-Z Systems, Palmer, PA, USA) until the loss of righting reflex and no response to toe pinch. After surgical site preparation and sterile draping, the rats were placed in the supine position on a thermostatically controlled heating pad set at 37 °C. A midline xiphopagic laparotomy was performed to expose the liver. The hepatic triad (hepatic artery, portal vein, and bile duct), supplying the left and median lobes, was occluded with atraumatic microvascular clips to induce partial warm liver ischemia (approximately 70% of liver mass). After 60 min of warm ischemia, the vascular clips were removed to initiate reperfusion. The abdominal incision was temporarily closed during the ischemic period and definitively sutured following the reperfusion initiation.

Finally, all rats were euthanized under deep isoflurane anesthesia, and blood samples from the inferior vena cava and ischemic liver lobes were collected, either fixed in 4% paraformaldehyde (PFA) for histological analysis or stored at −80 °C for biochemical and molecular assessments.

#### 2.5.2. Biochemical Plasma Analysis

##### Liver Injury Markers

Plasma alanine aminotransferase (ALT) and aspartate aminotransferase (AST) activities were assessed using a commercial kinetic test (Cat. #: 10025, and 11022, Biomaghreb Laboratories, Tunis, Tunisia) according to the manufacturer’s instructions.

##### Lipid Profile Assessment

Plasma total cholesterol (TC), triglycerides (TG), and high-density lipoprotein cholesterol (HDL-C) concentrations were determined using Cholestech LDX™ Lipid Profile GLU Cassette commercial kits (Cat. #: 14-531; Abbot Laboratories, Wiesbaden, Germany) according to the manufacturer’s instructions. Low-density lipoprotein cholesterol (LDL-c) was calculated using the Friedewald formula [18], and the LDL/HDL ratio was computed as an atherogenic index (AI).

#### 2.5.3. Oxidative Stress and Antioxidant Enzyme Activity Assessments

The liver tissues were homogenized (16% *w*/*v*) in cold 100 mM phosphate-buffered saline (PBS, pH 7.4) using a Polytron homogenizer (PT-MR 2100, KINEMATICA AG, Malters, Switzerland) maintained at 4 °C. The homogenates were centrifuged at 12,000 rpm for 25 min at 4 °C, and the supernatants were collected for subsequent analyses. The total protein content was determined using the Bradford method to normalize enzymatic activities.

##### Superoxide Dismutase (SOD) Activity

SOD activity was assessed according to the method of Marklund et al. [19], which is based on the SOD inhibition of pyrogallol autooxidation. In this assay, the rate of pyrogallol autoxidation is monitored spectrophotometrically by measuring the increase in absorbance at 420 nm, and SOD function is expressed as U/mg protein, where 1 unit is defined as the amount of enzyme required to cause 50% inhibition of pyrogallol autoxidation under the assay conditions.

##### Total Glutathione (GSH) Concentration

GSH concentration was determined according to the method described by Tietze (1969) [20], based on the reduction of 5,5′-dithiobis(2-nitrobenzoic acid) (DTNB) by GSH thiol groups (-SH), leading to the formation of 5-thio-2-nitrobenzoic acid (TNB). TNB formation was quantified by spectrophotometric measurement of absorbance at 412 nm [20].

##### Malondialdehyde (MDA) Assay

Lipid peroxidation was assessed by measuring MDA production using the thiobarbituric acid-reactive substances (TBARS) assay. Absorbance was measured spectrophotometrically at 530 nm. The results were expressed as nmol MDA per mg protein [21].

#### 2.5.4. Histological Analyses

The liver samples were fixed in 4% PFA (Cat. # 158127, Sigma-Aldrich Inc, St. Louis, MO, USA) in phosphate-buffered saline (Cat. # P4417, Sigma-Aldrich, Inc., St. Louis, MO, USA) 0.1 M (pH = 7.4) for 12–18 h. Subsequently, the specimens were dehydrated through a graded ethanol series, cleared in xylene, and embedded in paraffin (Cat. # 08-7910, Bio-Optica Milano S.p.a, Milan, Italy). Using a Leica RM2135 microtome, serial sections of 7 μm thickness were cut, mounted on gelatin-coated microscope slides, and left to dry for 24 h. Following the deparaffinization and rehydration, the sections were stained with hematoxylin and eosin (Cat. #05-M0612 and Cat. #05-M10002 Bio-Optica Milano S.p.a, Milan, Italy). The morphological evaluation and image capture were performed using a Leica DMRB light microscope equipped with a Leica DFC7000 T camera (Leica Microsystems GmbH, Wetzlar, Germany), as described in [22]. The degree of hepatic steatosis was quantified based on the liver steatosis score: S0 (absent, <5%), S1 (mild, 5–33%), S2 (moderate, 33–66%), and S3 (severe, >66%) [23,24]. The severity of liver injury was graded with Suzuki’s criteria, where sections were scored from 0 to 4 for sinusoidal congestion, vacuolization of hepatocyte cytoplasm, and parenchymal necrosis.

#### 2.5.5. Immunofluorescence Analysis of IL1-B and *TNF-α*

To evaluate the expression of the pro-inflammatory cytokines interleukin-1 beta (IL-1β) and tumor necrosis factor alpha (*TNF-α*), serial sections were washed in working buffer (Tris–HCl buffer 0.05 M, pH 7.5) containing 0.1% bovine serum albumin and 0.2% Triton-X 100. Non-specific binding sites were blocked by incubating the slides with 25% fetal bovine serum (F7524 Sigma-Aldrich, St. Louis, MO, USA) for 30 min at room temperature. The sections were then incubated overnight at 4 °C with the primary antibodies against IL-1β and TNF-α (see Table 1). After rinsing with the working buffer, the appropriate fluorophore-conjugated secondary antibodies were applied for 90 min (see Table 1). Finally, the sections were mounted using Fluoromount Aqueous Mounting Medium (Sigma-Aldrich, Inc., St. Louis, MO, USA. Cat. #F4680) [25,26]. To ensure specificity, negative controls were conducted by omitting the primary antibody, which resulted in no detectable immunostaining (Supplementary Materials, Figure S1).

#### 2.5.6. Quantitative Real-Time PCR (qRT-PCR)

The total RNA was extracted from frozen liver tissues using TRIzol^®^ reagent (Invitrogen, Carlsbad, CA, USA), and mRNA purity and integrity were assessed through agarose gel electrophoresis and spectrophotometry (260/280 nm absorbance ratio). Subsequently, two micrograms of the total RNA were reverse-transcribed into complementary DNA (cDNA) using the M-MLV RT enzyme kit (Thermo Scientific Invitrogen™, Illkirch, France). Quantitative RT-PCR was performed using SYBR Green Supermix (Cat. No. 1725270, Bio-Rad Laboratories, Hercules, CA, USA) on a Step-One Real-Time PCR system (Thermo Fisher Scientific, Landsmeer, The Netherlands). The amplification protocol consisted of an initial denaturation step followed by 40 cycles (95 °C for 15 s, 58 °C for 30 s, and 72 °C for 40 s), 95 °C for 15 s toward the end of the amplification phase). The melting curve analysis was conducted to verify amplification specificity.

The expression levels of inflammatory markers (*TLR4*, *NF-κB*, *TNF-α*, and *IL-1β*), inflammasome-related genes (*NLRP3*, *GSDMD*, *IL-18*), antioxidant-related genes (*GPx*, *HO-1*), and metabolic regulators (*AMPK*, *SREBP-1c*) was calculated using the 2^−ΔΔCt^. The data were normalized against housekeeping genes (GAPDH or β-actin) and the specific primer sequences in Table 2.

### 2.6. Statistical Analysis

The statistical analyses were conducted using IBM SPSS Statistics for Windows version 22 (IBM Corp, Armonk, NY, USA) and GraphPad Prism, version 8.0.1, for Windows (GraphPad Software, San Diego, CA, USA). Data normality and homogeneity of variance were checked prior to the analysis. Pairwise comparisons were assessed using a one-way analysis of variance (ANOVA) followed by Tukey’s post hoc test when assumptions of normality and homogeneity were satisfied. When these assumptions were violated, Welch’s ANOVA followed by Games–Howell post hoc test was applied. The changes in body weight and insulin tolerance test were analyzed using a linear mixed-effects model, with group and time as fixed effects, and incorporating repeated measures within subjects. The histological data, including Suzuki and steatosis grade, were analyzed using the Kruskal–Wallis test followed by Dunn’s multiple comparisons. The results are presented as mean ± SD (or SEM for model estimates), with the threshold for statistical significance set at *p* < 0.05.

## 3. Results

### 3.1. High-Fat Diet Induces Metabolic Alterations

**Figure 2:**
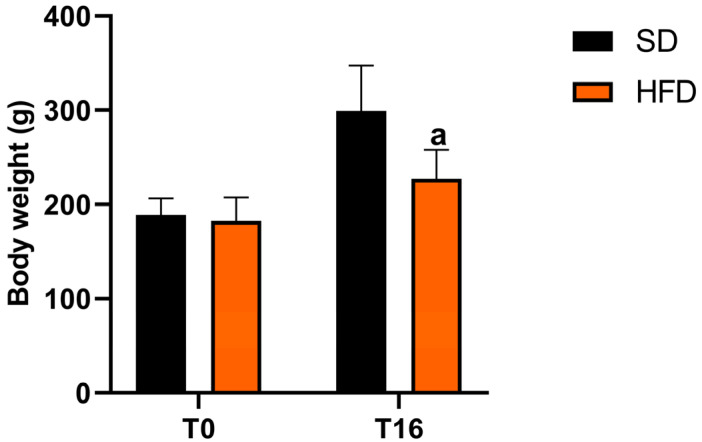
Body weight at T0 and T16 in rats fed a standard diet (Sd) (*n* = 12) or high-fat diet (HFD) (*n* = 18). The data are presented as estimated marginal means ± SEM from the linear mixed-effects model. a indicates a significant difference compared with the Sd group at the same time point (*p* < 0.05). Superscript letter a indicates a statistically significant difference versus the Sd group (*p* < 0.05).

Baseline body weight did not differ between rats fed the standard diet (188.9 ± 17.4 g) and those fed the high-fat diet (183.2 ± 24.0 g) (t(27) = 0.71, *p* = 0.482). Subsequent changes were evaluated using a linear mixed-effects model, with time and diet as fixed effects and subject as a random factor. A significant effect of time was observed (F (1,27) = 265.59, *p* < 0.001), indicating an overall increase in body weight during the study. A significant effect of the diet group was also detected (F (1,27) = 11.72, *p* = 0.002). Importantly, a significant time × group interaction was found (F (1,27) = 42.10, *p* < 0.001), demonstrating that weight gain differed between the two diet groups. Indeed, rats fed the standard diet increased from 188.9 g to 299.0 g (+110.1 g), whereas rats fed the high-fat diet increased from 183.2 g to 230.5 g (+47.4 g) (Figure 2).

**Figure 3:**
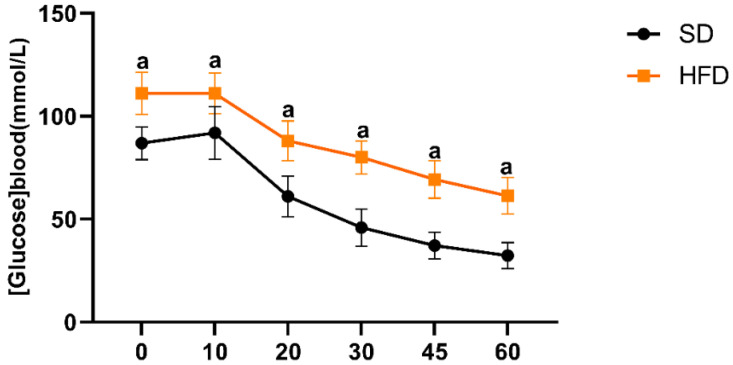
Time-dependent changes in blood glucose levels following intraperitoneal injection of insulin (0.5 IU/kg body weight) in rats fed a high-fat diet compared with those fed a standard diet (*n* = 12 for Sd and *n* = 18 for HFD). Insulin tolerance testing was performed after a 5 h fast, and blood glucose concentrations were measured at 0, 10, 20, 30, 45, and 60 min. Data are presented as mean ± SEM. Statistical differences were assessed using the linear mixed-effects model with Bonferroni’s post hoc test. Superscript letter a indicates a statistically significant difference versus the standard diet (*p* < 0.05).

An insulin tolerance test (ITT) was performed to assess baseline insulin sensitivity in the animals. As shown in Figure 3, intraperitoneal insulin injection of 0.5 IU/kg led to a reduction in blood glucose concentration over time in both groups, as indicated by the linear mixed-effects model for the time effect (F (5,19) = 133.61, *p* < 0.001). Moreover, the analysis revealed significant effects of the group (F (1,19) = 60.34, *p* < 0.001) and the group × time interaction (F (5,19) = 3.13, *p* = 0.031). Post hoc Bonferroni comparisons showed that HFD rats exhibited significantly higher blood glucose levels and thus reduced insulin sensitivity compared to standard diet-fed rats at all points (*p* < 0.01).

### 3.2. High-Fat Diet Changes Lipid Profile

**Figure 4:**
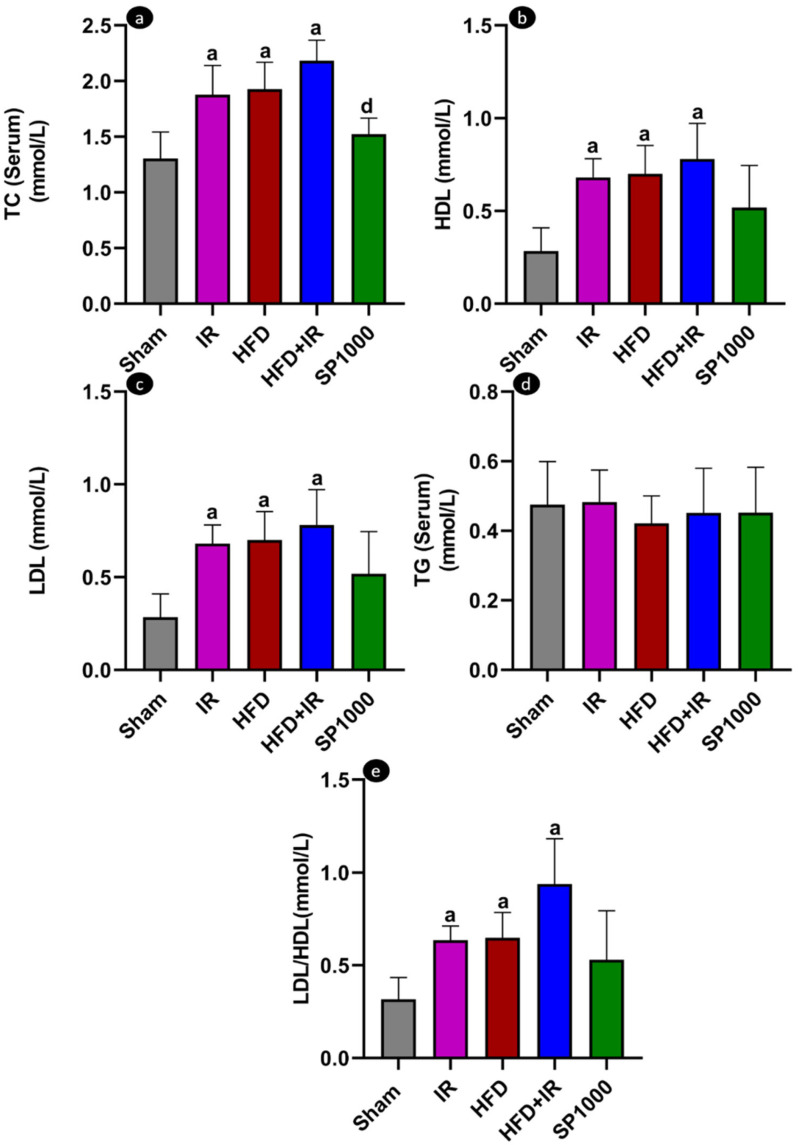
The effects of high-fat diet, ischemia–reperfusion, and spirulina supplementation on the lipid profile parameters. (**a**) Total cholesterol (TC), (**b**) high-density lipoprotein (HDL), (**c**) low-density lipoprotein (LDL), (**d**) triglycerides (TG), and (**e**) LDL/HDL ratio. The data are presented as mean ± SD (*n* = 6 rats per group). The statistical analysis was performed using one-way ANOVA followed by Tukey’s post hoc test for TC, HDL, LDL, and TG data (*p* < 0.05). LDL/HDL ratio data were analyzed using Welch ANOVA followed by Games–Howell’s post hoc test (*p* < 0.05). Statistical significance is indicated as follows: a vs. sham and d vs. HFD + IR.

Serum lipid parameters were evaluated to assess the effects of dietary intervention, IR, and spirulina supplementation (Figure 4a–e).

Feeding rats a high-fat diet (HFD) for 19 weeks resulted in significant alterations in lipid parameters compared with the sham group (*p* < 0.05). The same pattern was observed in HFD-fed rats exposed to IR (HFD + IR group), showing additional increases in TC (F (4,20) = 12.12, *p* < 0.0001, η^2^ = 0.708), LDL-C (F (4,17) = 6.304, *p* < 0.05, η^2^ = 0.597), and the LDL/HDL ratio compared with the HFD group; however, these differences did not reach statistical significance. Spirulina supplementation (HFD + IR + SP1000) was associated with partial improvement in lipid parameters compared with the untreated HFD + IR group, although these changes did not reach statistical significance. Triglyceride (TG) levels were not significantly different among any of the groups (F (4,18) = 0.2193, *p* = 0.924, η^2^ = 0.046).

### 3.3. Spirulina Modulates Liver Injury in HFD Rats Subjected to IR Injury: Alanine (ALT) and Aspartate (AST) Aminotransferase Activities

**Figure 5:**
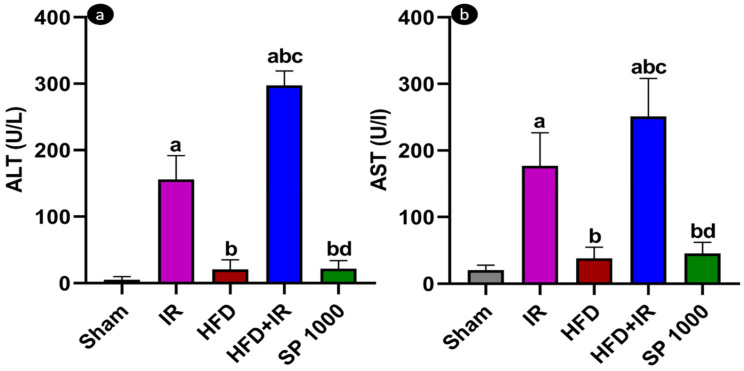
The evaluation of liver injury markers: (**a**) plasma alanine aminotransferase (ALT) and (**b**) aspartate aminotransferase (AST) levels in the sham group, high-fat diet (HFD), ischemia–reperfusion (IR), HFD + IR, and SP 1000-treated groups after 1 h of ischemia followed by 24 h of reperfusion. The data are expressed as mean ± SD (*n* = 6 rats per group). The statistical analysis was performed using one-way ANOVA followed by Tukey’s post hoc test. The differences were considered statistically significant at *p* < 0.05. Statistical significance is indicated as follows: a vs. sham; b vs. IR; c vs. HFD; and d vs. HFD + IR.

After 24 h of reperfusion, serum ALT was quantified in the different experimental groups. Compared with the ALT baseline level of the sham group, the IR group showed a significant increase (F (4,18) = 154.2, *p* < 0.0001, η^2^ = 0.972). Indeed, the IR group’s ALT activity was 155.92 U/L, while that of the sham group was 5.075 U/L. Moreover, ALT activity in the HFD + IR was about twice that of the IR group (*p* < 0.0001) (297.5 U/L) and more than 15 times that of the HFD group. Finally, the ALT level in the SP1000 group was statistically lower than those of the HFD + IR (*p* < 0.0001) and IR (*p* < 0.0001) groups and comparable to that of the sham group (*p* < 0.75) (Figure 5a).

Similarly, AST activity was significantly higher in the IR group than in the sham group (F (4,19) = 38.56, *p* < 0.0001, η^2^ =0.89). Furthermore, HFD + IR AST activity was significantly higher than that of the IR group (*p* < 0.05) (Figure 5b). The pretreatment of rats with spirulina at a dose of 1000 mg/kg BW/day significantly attenuated both enzyme activities (*p* < 0.0001).

### 3.4. Spirulina Modulates Liver Architecture in Spirulina-Treated HFD Rats Subjected to IR Injury

**Figure 6:**
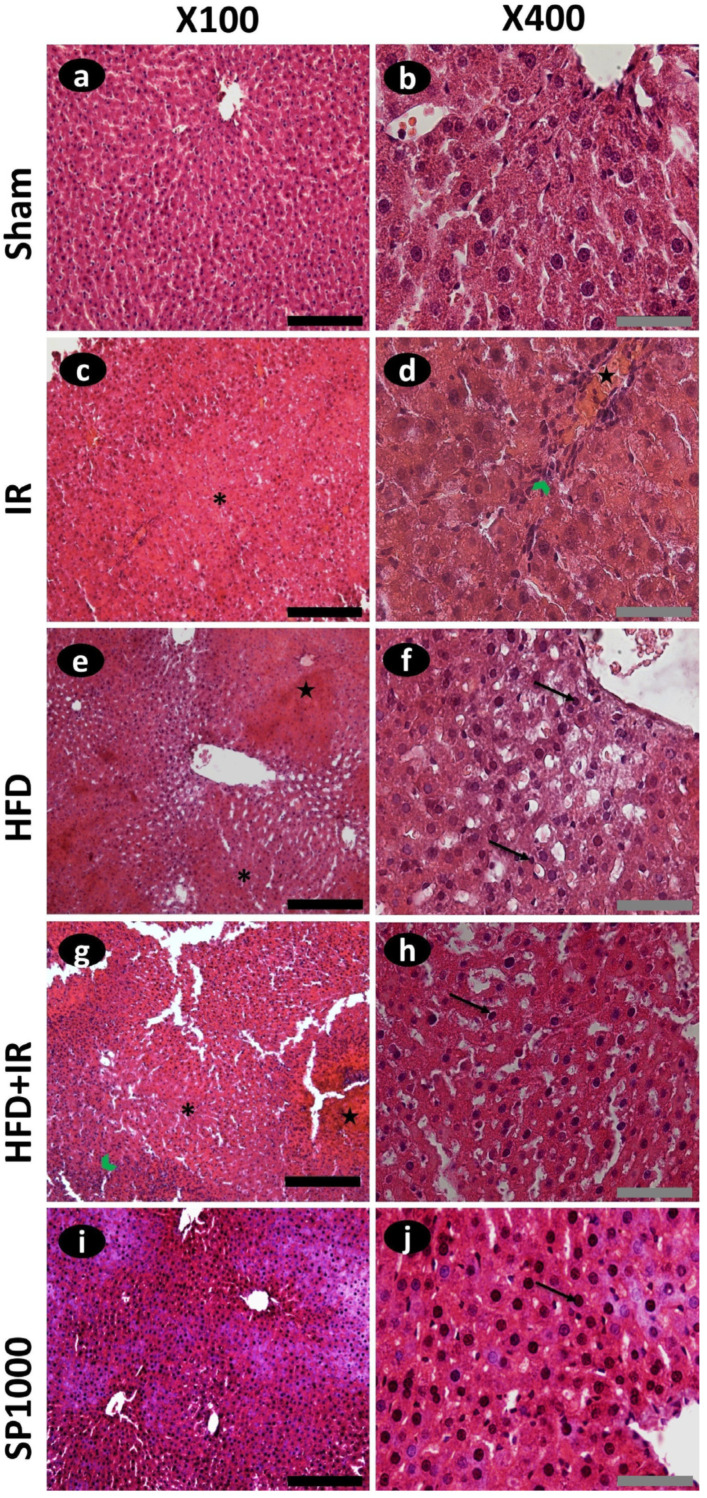
Photomicrographs of hematoxylin–eosin-stained liver tissue of the (**a**,**b**) sham group, (**c**,**d**) IR group, (**e**,**f**) HFD group, (**g**,**h**) HFD + IR group, and (**i**,**j**) SP1000 group. Cell infiltration (green chevron arrows). Necrosis (black asterisk). Congestion (black stars). Pyknotic nuclei (black arrows). The black scale bar represents 200 μm, and the gray scale bar represents 50 μm.

Representative liver sections were examined to evaluate morphological changes across experimental groups (Figure 6a–j). Sham liver had a normal parenchyma composed of single-cell thick anastomosing plates of acidophilic polygonal hepatocytes, exhibiting a uniform shape with well-defined cell boundaries, clear cytoplasm, and large central vesicular nuclei (Figure 6a,b). In contrast, histological examination of liver tissues from IR rats revealed alterations in hepatic architecture characterized by darkly stained pyknotic nuclei, sinusoidal congestion, hepatocellular swelling, and focal inflammatory cell infiltration (Figure 6c,d).

To objectively quantify the impact of high-fat diet and spirulina treatment on hepatic lipid accumulation, liver steatosis was graded according to the percentage of hepatocytes exhibiting fatty infiltration: S0 (<5%), S1 (5–33%), S2 (33–66%), and S3 (>66%).

**Figure 7:**
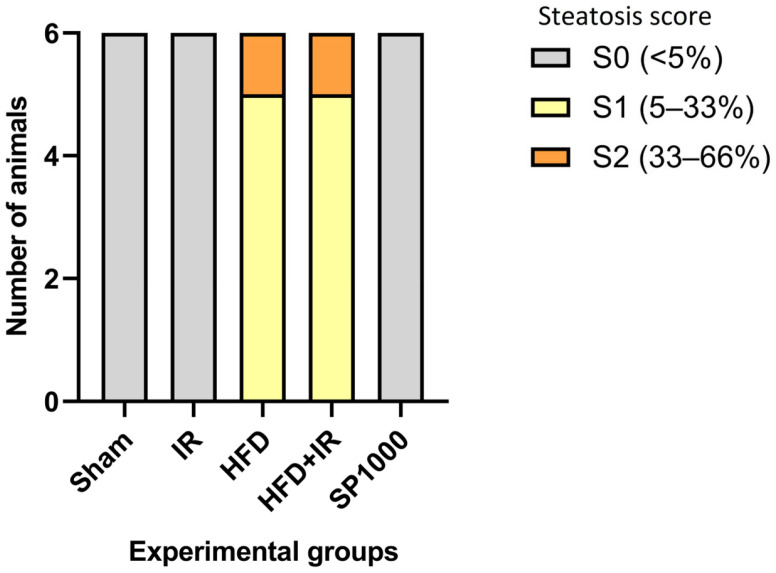
The distribution of hepatic steatosis grades across experimental groups. Hepatic steatosis was evaluated histologically and graded according to the percentage of hepatocytes containing lipid droplets: S0 (<5%), S1 (5–33%), S2 (33–66%), and S3 (>66%). The stacked bars represent the number of animals in each steatosis grade within each experimental group (sham, IR, HFD, HFD + IR, and SP1000). Steatosis was absent in the sham, IR, and SP1000 groups (all S0), whereas animals in the HFD and HFD + IR groups predominantly exhibited mild steatosis (S1) with occasional moderate steatosis (S2).

Steatosis was entirely absent (grade S0) in both the sham and IR groups (Figure 7). The HFD-fed rats’ livers displayed marked histological alterations, with distorted hepatocyte arrangement, and predominantly exhibited mild steatosis (S1), with some animals showing moderate steatosis (S2), and inflammatory cell infiltration (Figure 6e,f and Figure 7). These alterations were more marked in the HFD + IR group, where increased steatosis, sinusoidal congestion, and inflammatory cell presence were detected compared with the animals from the IR group. Notably, spirulina supplementation (SP1000 group) markedly attenuated these histological alterations. According to the steatosis scoring system, animals in the SP1000 group were classified as (grade S0: <5% steatosis), indicating the absence of detectable steatosis, with a hepatic architecture that remained comparable to the sham group (Figure 7, Tables S2 and S3).

**Figure 8:**
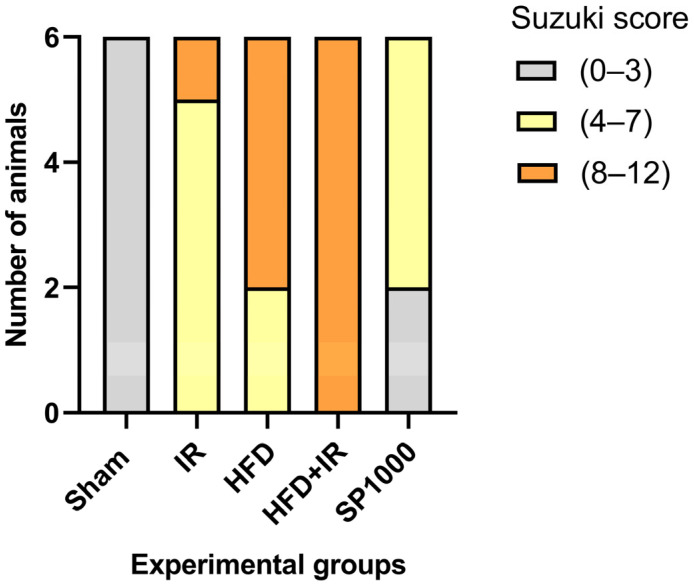
The distribution of hepatic injury severity based on Suzuki scoring in different experimental groups (sham, IR, HFD, HFD + IR, and SP1000). Bars represent the number of animals (*n* = 6 per group) categorized into Suzuki score ranges: 0–3 (minimal injury), 4–7 (moderate injury), and 8–12 (severe injury). The sham group exhibited minimal histological damage, whereas the HFD and HFD + IR groups showed a higher proportion of animals with severe liver injury. The treatment with SP1000 reduced the severity of hepatic injury compared with the HFD + IR group. The statistical analysis of Suzuki scores was performed using the Kruskal–Wallis test followed by pairwise comparisons, with significance set at *p* < 0.05.

Suzuki histological scores (Figure 8) were compared among groups using the Kruskal–Wallis test followed by Dunn’s post hoc pairwise comparisons. A significant difference in liver injury scores was observed between the groups. The pairwise analysis demonstrated that the HFD group showed significantly higher Suzuki scores compared with the sham group (adjusted *p* = 0.004). Similarly, the HFD + IR group exhibited significantly greater liver injury than the sham group (adjusted *p* < 0.001). Furthermore, SP1000 treatment significantly reduced liver injury compared with the HFD + IR group (adjusted *p* = 0.030) (Tables S4 and S5).

### 3.5. Spirulina Modulates Hepatic Oxidative Stress in HFD Rats After IR Injury

**Figure 9:**
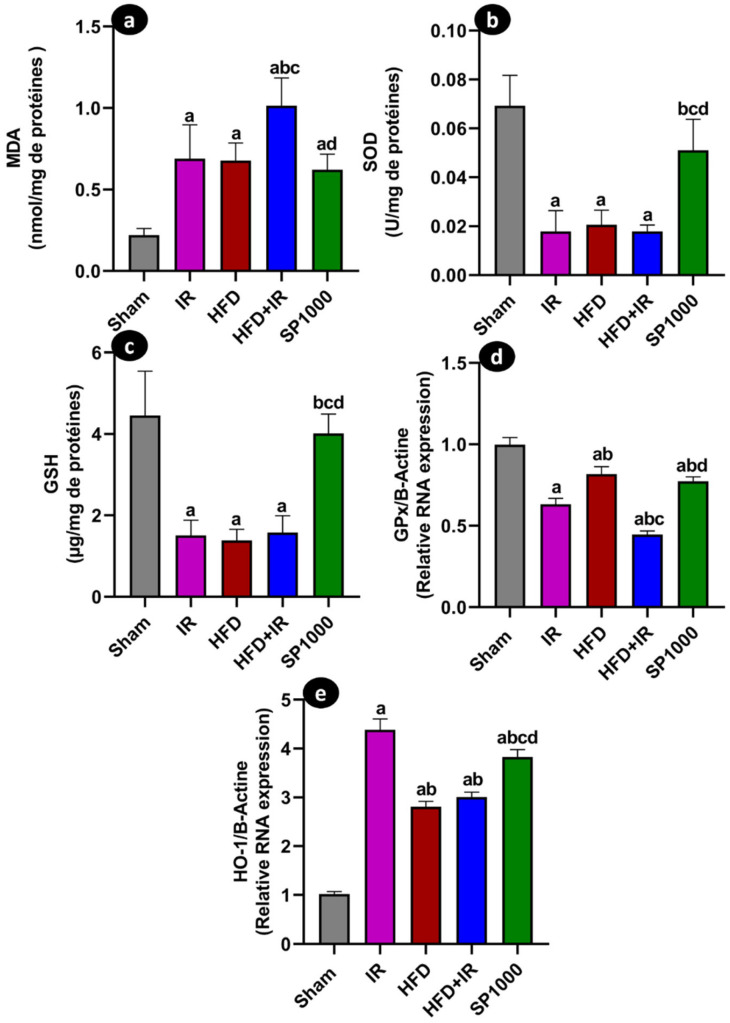
The effects of spirulina supplementation on hepatic oxidative stress following IR injury in steatotic livers. (**a**) Malondialdehyde (MDA) concentration, (**b**) superoxide dismutase (SOD) activity, (**c**) total glutathione (GSH) concentration, (**d**) glutathione peroxidase (*GPx*) mRNA expression, and (**e**) heme oxygenase-1 (*HO-1*) mRNA expression in the sham, IR, HFD, HFD + IR, and SP1000 groups. The data are presented as mean ± SD (*n* = 6 rats per group). The statistical analysis was performed using the one-way ANOVA followed by Tukey’s post hoc test for MDA, SOD, *GPx*, and *HO-1* data (*p* < 0.001). The GSH data were analyzed using Welch ANOVA followed by Games–Howell’s post hoc test (*p* < 0.05). Statistical significance is indicated as follows: a vs. sham; b vs. IR; c vs. HFD; and d vs. HFD + IR.

Hepatic oxidative stress markers were assessed by measuring lipid peroxidation and antioxidant parameters (Figure 9a–e). MDA concentration (Figure 9a) was significantly increased in the IR group compared with the sham group (*p* < 0.05). A further increase was observed in the HFD + IR group relative to the IR group (*p* < 0.05). In the HFD + IR + SP1000 group, MDA concentrations decreased and were significantly lower than in the untreated HFD + IR group (*p* < 0.05) (F (4,19) = 23.3, *p* < 0.001, η^2^ = 0.831). SOD activity (Figure 9b) was significantly lower in the IR group than in the sham animals (*p* < 0.05). A more pronounced reduction in enzyme activity was observed in rats fed a HFD prior to the IR induction (HFD + IR group). In contrast, SOD activity was significantly higher in rats treated with SP1000 than in the untreated HFD + IR group (*p* < 0.05) (F (4,17) = 28.96, *p* < 0.0001, η^2^ = 0.872). Consistently, hepatic GSH levels (Figure 9c) were significantly lower in the IR group than in the sham group (*p* < 0.05). The GSH levels were further reduced in the HFD + IR group. SP1000 supplementation resulted in significantly higher GSH levels compared with the untreated HFD + IR group (*p* < 0.05) (Welch (4,8.25) = 23.12, *p* < 0.001).

Gene expression analysis of *GPx* (Figure 9d) revealed significantly reduced mRNA levels in the IR group compared with the sham animals (*p* < 0.05), with a further decrease in the HFD + IR group. In the HFD + IR + SP1000 group, *GPx* expression was augmented and was significantly higher than in the HFD + IR group (*p* < 0.05) (F (4,20) = 164.1, *p* < 0.0001, η^2^ = 0.97).

A similar pattern was observed for *HO-1* mRNA expression (Figure 9e). We observed a significant decrease in the IR and HFD + IR groups compared with the sham animals (*p* < 0.05). The SP1000 treatment was associated with significantly higher *HO-1* expression compared with the untreated HFD + IR group (*p* < 0.05) (F (4,20) = 426.1, *p* < 0.0001, η^2^ = 0.988).

### 3.6. Spirulina Attenuates Hepatic Inflammatory Signaling at mRNA and Protein Levels

**Figure 10:**
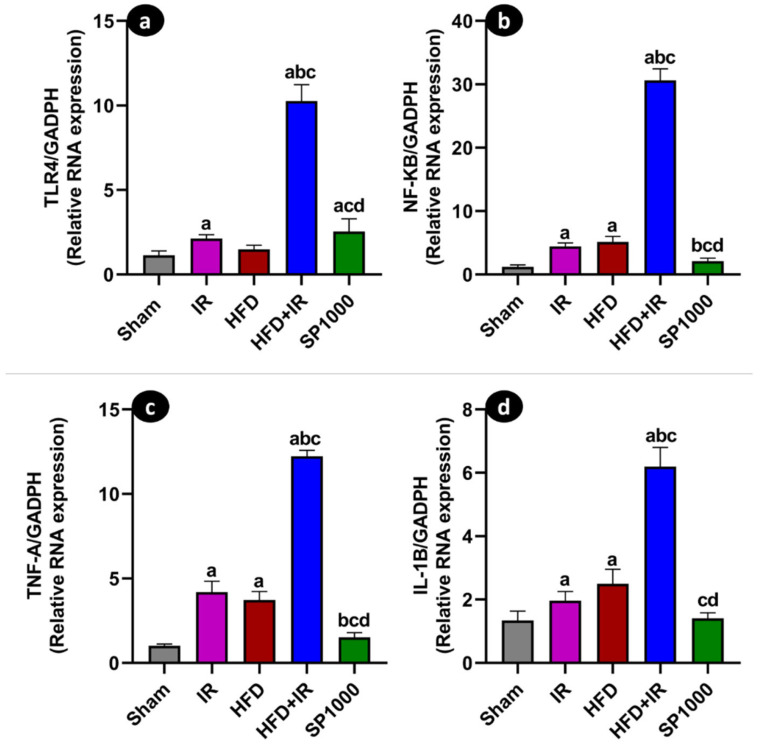
The effect of spirulina treatment on the inflammatory gene expression. (**a**) Toll-like receptor 4 (*TLR4*), (**b**) nuclear factor kappa B (*NFKB*), (**c**) tumor necrosis factor alpha (*TNF-A*), and (**d**) interleukin 1 beta (*IL1-B*) were assessed through RT-PCR on liver tissue of the sham, IR, HFD, HFD + IR and SP1000 groups. The data are presented as mean ± SD (*n* = 6 rats per group). The statistical differences were assessed using the one-way ANOVA followed by Tukey’s post hoc test (*p* < 0.001 for all genes). Statistical significance is denoted as follows: a vs. sham; b vs. IR; c vs. HFD; and d vs. HFD + IR.

The hepatic mRNA expression of inflammatory markers (*TLR4*, *NF-κB*, *TNF-α*, and *IL-1β*) was assessed through quantitative RT-PCR (Figure 10a–d). All four markers exhibited a comparable pattern across the experimental groups. Compared with the sham group, *TLR4* (Figure 10a), *NF-κB* (Figure 10b), *TNF-α* (Figure 10c), and *IL-1β* (Figure 10d) mRNA levels were significantly increased in both the IR and HFD groups (*p* < 0.05) (F (4,25) = 264.6, *p* < 0.0001, η^2^ = 0.977, F (4,20) = 820.3, *p* < 0.0001, η^2^ = 0.994, F (4,35) = 953.3, *p* < 0.0001, η^2^ = 0.991, F (4.30) = 184.2, *p* < 0.0001, η^2^ = 0.961), respectively. The expression levels were further elevated in the HFD + IR group, which consistently showed the highest values among all groups (*p* < 0.05). In contrast, SP1000 supplementation (HFD + IR + SP1000) was associated with significantly lower expression levels of *TLR4*, *NF-κB*, *TNF-α*, and *IL-1β* compared with the untreated HFD + IR group (*p* < 0.05).

**Figure 11:**
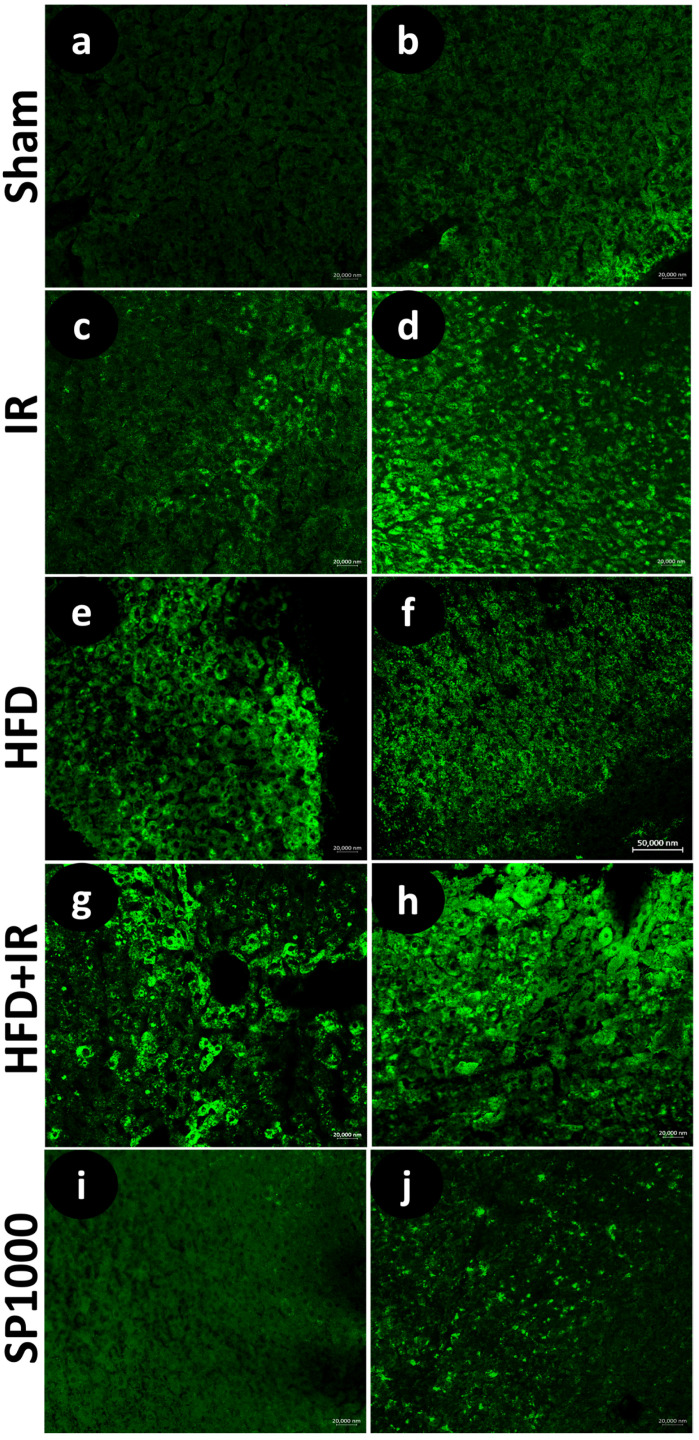
The immunofluorescence photomicrographs of TNF-A (**a**,**c**,**e**,**g**,**i**) and IL1-B (**b**,**d**,**f**,**h**,**j**) in liver tissue of the (**a**,**b**) sham group, (**c**,**d**) IR group, (**e**,**f**) HFD group, (**g**,**h**) HFD + IR group, and (**i**,**j**) SP1000 group. Magnification: 20×.

These findings were further confirmed by the immunofluorescence analysis (Figure 11a–j), which revealed a visibly stronger immunofluorescent staining for both TNF-α (Figure 11a–i) and IL-1β (Figure 11b–j) immunoreactivity in livers exposed to IR and HFD. Moreover, the combined exposure to HFD and IR (HFD + IR group) was associated with a further apparent increase in fluorescence labeling, suggesting enhanced inflammatory responses in liver tissue under combined exposure increased immunolabeling. In contrast, the spirulina group (SP1000) showed a visibly lower TNF-α and IL-1β immunofluorescent signal compared with the HFD + IR group. These observations are based on the qualitative assessment of representative images, as fluorescence intensity was not quantitatively measured.

### 3.7. Spirulina Reduces Inflammasome Activation and Pyroptosis-Related Gene Expression

**Figure 12:**
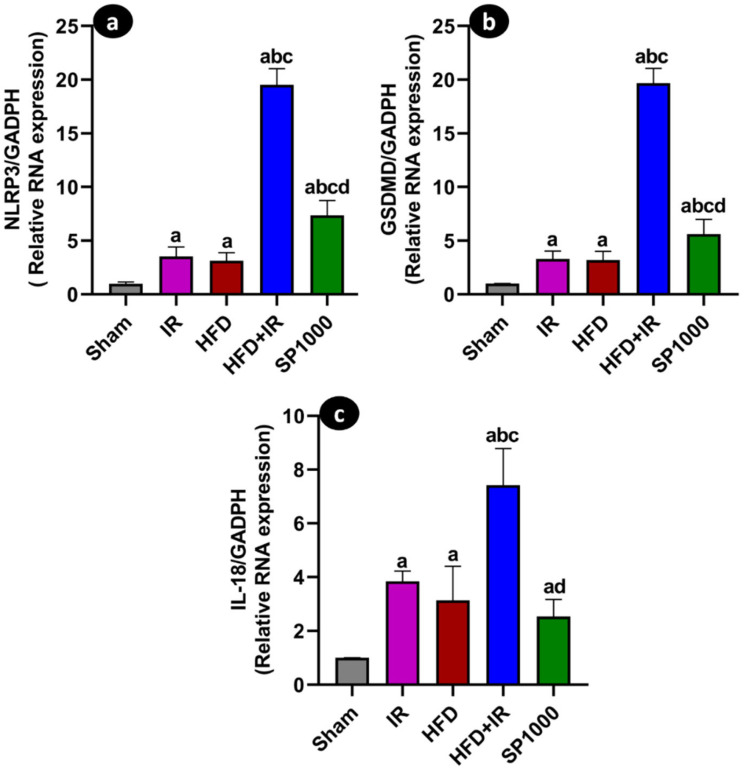
The effects of spirulina supplementation on inflammasome and pyroptosis-related gene expression following ischemia–reperfusion (IR) injury in steatotic liver. Hepatic mRNA expression of (**a**) NOD-like receptor protein 3 (*NLRP3*), (**b**) gasdermin D (*GSDMD*), and (**c**) interleukin-18 (*IL-18*) assessed through qRT-PCR in the sham, IR, HFD, HFD + IR, and SP1000 groups. The data are presented as mean ± SD (*n* = 6 rats per group). The statistical differences were assessed using the one-way ANOVA followed by Tukey’s post hoc test (*p* < 0.05). Statistical significance is indicated as follows: a vs. sham; b vs. IR; c vs. HFD; and d vs. HFD + IR.

We evaluated the inflammasome activation through *NLRP3* expression, followed by the assessment of its downstream effector cytokine, *IL-18*, and the pyroptotic executor, *GSDMD* (Figure 12a–c). All three markers exhibited a consistent expression profile across experimental groups. Compared with the sham group, the IR and HFD groups showed a significant increase in *NLRP3* (Figure 12a), *IL-18* (Figure 12c), and *GSDMD* (Figure 12b) transcripts in (F (4,25) = 298.8, *p* < 0.0001, η^2^ = 0.975; F (4,25) = 42.49, *p* < 0.0001, η^2^ = 0.872; F (4,25) = 336.7, *p* < 0.0001, η^2^ = 0.981), respectively. The exposure of HFD rats to IR further elevated gene expression, with this group displaying the highest mRNA levels among all experimental groups (*p* < 0.05). In contrast, SP1000 supplementation was associated with a significant reduction in *NLRP3*, *IL-18*, and *GSDMD* expression compared with the untreated HFD + IR group (*p* < 0.05). The expression levels in rats treated with SP1000 were markedly lower than those observed in the HFD + IR group and tended to approach the levels assessed in the sham group.

### 3.8. Spirulina Modulates Lipid Metabolism-Related Gene Expression

**Figure 13:**
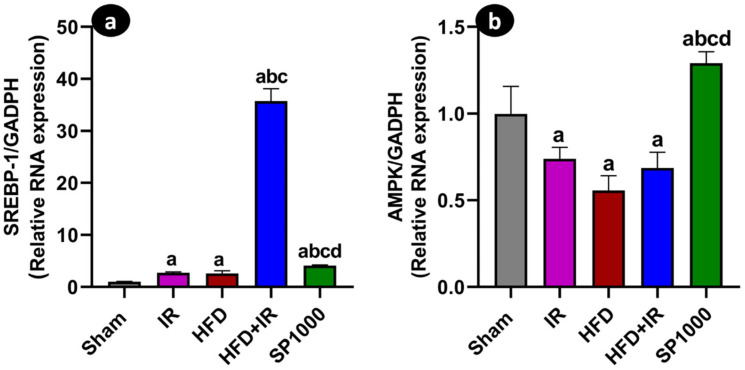
The effects of spirulina supplementation on lipid metabolism-related gene expression following ischemia–reperfusion (IR) injury in steatotic liver. Hepatic mRNA expressions of (**a**) sterol regulatory element-binding protein-1c (*SREBP-1*) and (**b**) *AMPK* were assessed through qRT-PCR in the sham, IR, HFD, HFD + IR, and SP1000 groups. The data are presented as mean ± SD (*n* = 6 rats per group). Statistical analysis was performed using the Welch ANOVA followed by Games–Howell’s post hoc test for *SREBP-1* (*p* < 0.05), and the one-way ANOVA followed by Tukey’s post hoc test for *AMPK* (*p* < 0.05). Statistical significance is indicated as follows: a vs. sham; b vs. IR; c vs. HFD; and d vs. HFD + IR.

The hepatic mRNA expression of lipid metabolism-related markers, including *SREBP-1c* and *AMPK*, was assessed through quantitative RT-PCR (Figure 13a,b).

The *SREBP-1c* expression was significantly increased in the IR and HFD groups compared with the sham group (*p* < 0.05). A significant increase was noted in the HFD + IR group, which exhibited the highest expression levels among all groups (*p* < 0.05). In contrast, supplementation with SP1000 in rats was associated with a significant reduction in the *SREBP-1c* expression compared with the untreated HFD + IR group (*p* < 0.05).

Conversely, the *AMPK* mRNA expression displayed an opposite pattern. Compared with the sham group, *AMPK* levels were significantly decreased in the IR, HFD, and HFD + IR groups (*p* < 0.05). In rats receiving SP1000, the *AMPK* expression was significantly higher than in all groups (*p* < 0.05) (F (4,20) = 43.15, *p* < 0.01, η^2^ = 0.896).

## 4. Discussion

The persistent gap between liver transplantation demand and graft availability remains a major challenge in hepatology and transplantation [27]. In parallel, the growing prevalence of non-alcoholic fatty liver disease (NAFLD) has increased the proportion of donor grafts exhibiting hepatic steatosis [28]. However, hepatic steatosis is a key determinant of susceptibility to IR injury [29]. Steatotic livers typically develop a more pronounced oxidative and inflammatory response after reperfusion, which induces greater hepatocellular damage and poorer outcomes [10]. Therefore, there is a need to explore strategies that can be implemented to attenuate IR injury in fatty livers.

Spirulina (*Arthrospira* spp.) is a nutrient-rich cyanobacterium that contains phenolic compounds, phycobiliproteins, especially phycocyanin, and carotenoids. These compounds contribute to its wide range of biological activities, notably antioxidant, anti-inflammatory, immunostimulatory, and hepatoprotective properties [30,31,32,33,34,35]. Based on these findings, we investigated whether preconditioning rats with spirulina could alleviate IR injury in a model of diet-induced hepatic steatosis subjected to partial warm ischemia followed by reperfusion.

The metabolic characterization performed prior to ischemia–reperfusion induction confirmed that the dietary intervention produced distinct physiological adaptations between groups. Both the standard diet and the high-fat diet-fed animals exhibited an overall increase in body weight during the 16-week feeding period, reflecting normal growth in young adult rats. However, the extent of weight gain differed significantly between the animals from the two diet groups. Indeed, rats fed the standard diet showed greater body weight gain than those receiving the high-fat diet. The lower body weight gain observed in the HFD group cannot be attributed to reduced feed intake or health problems. Rats consumed the full daily amount of feed provided and did not show any signs of reduced palatability. Thus, spontaneous caloric restriction is also unlikely, since the estimated daily energy intake of the HFD group was higher than that of the Sd group (≈98.8 kcal/rat/day in HFD vs. ≈59.4 kcal/rat/day in Sd-fed rats). In addition, the protein content of the diet (slightly above 13%) falls within the recommended range for adult maintenance diets (12–16%), suggesting that inadequate protein intake was unlikely to limit growth [15,36]. Moreover, the animals were monitored throughout the experiment and did not exhibit clinical signs of illness. This apparently paradoxical finding has been reported in some experimental models and suggests that the metabolic effects of a high-fat diet are not solely determined by the caloric intake. High-fat feeding is known to induce metabolic dysregulation, including insulin resistance, altered lipid handling, and impaired mitochondrial energy metabolism, which can influence energy expenditure and growth patterns independently of the caloric intake [37,38,39]. Consistent with this interpretation, the HFD-fed rats showed a significant reduction in insulin sensitivity compared with standard diet-fed rats, consistent with impaired systemic insulin sensitivity [40,41]. Together with the histological evidence of hepatic steatosis, these findings confirm that the high-fat diet successfully induced a metabolically compromised phenotype that closely resembles non-alcoholic fatty liver disease.

To assess the potential hepatoprotective effects of spirulina, the plasma ALT and AST activities were quantified in a rat model of diet-induced steatosis subjected to 70% partial hepatic IR injury, confirming the hepatocellular injury state. IR increased significantly both enzyme activities, an effect that was further exacerbated in the livers of the HFD-fed rats. These findings are in line with previous studies showing that steatosis reduces hepatic tolerance to IR [10]. Notably, the pretreatment of rats with spirulina significantly attenuated the ALT and AST levels in HFD-exposed rats subjected to IR. This is consistent with prior studies in which rats fed diets enriched with 5–10% spirulina showed reductions in the ALT and AST levels [42]. This suggests that spirulina may exert a protective effect against liver cell injury, an effect likely mediated, at least in part, by phycocyanin [43]. These biochemical findings were confirmed by the histological observations. Indeed, the sham livers exhibited preserved architecture, whereas the IR livers showed sinusoidal congestion, hepatocellular swelling, and focal inflammatory cell infiltration. High-fat feeding induced prominent cytoplasmic lipid vacuolization, and the combination of HFD and IR was associated with more extensive architectural disruption, congestion, and inflammatory cell presence. In contrast, the preconditioning of rats with spirulina showed a more preserved hepatic architecture, with a feature approaching those observed in the sham group.

NAFLD is well-known to primarily result from insulin resistance and is closely associated with hepatic lipid accumulation and metabolic dysregulation [44]. In insulin-resistant individuals, increased lipolysis leads to the release of free fatty acids, which accumulate in the liver and contribute to hepatic steatosis [45,46]. Insulin signaling is known to promote hepatic de novo lipogenesis in part through the transcriptional activation of sterol regulatory element-binding protein-1c (*SREBP-1c*), notably via liver X receptor (LXR)-dependent mechanisms [47]. In the current study, indeed, a marked upregulation of hepatic *SREBP-1c* mRNA in the HFD and IR conditions was recorded, while HFD + IR had the highest expression levels. This supports an enhanced lipogenic drive when steatosis and reperfusion injury coexist [48]. The spirulina pretreatment, however, reduced the *SREBP-1c* expression toward the sham profile. Those findings are in accordance with the results of Zhu et al. (2023), where *SREBP-1c* and subsequent transcription of its downstream lipogenic target genes were upregulated in cows with severe fatty liver [49], while the spirulina pretreatment had restored the *SREBP-1c* expression toward basal levels. In parallel, AMPK is a key energy sensor that restrains hepatic lipogenesis. It directly inhibits the SREBP activity and limits SREBP-driven transcriptional programs [50]. Consistent with this framework, the *AMPK* expression was reduced in the groups exposed to HFD and/or IR, whereas the spirulina pretreatment restored the *AMPK* expression and concomitantly reduced *SREBP-1c*. This may indicate a coordinated modulation of genes involved in lipid metabolism in the treated group [50]. The increase in *AMPK* coupled with a reduction in *SREBP-1c* points to a metabolically protective effect that may contribute to improved tolerance of steatotic livers to IR after the spirulina treatment [51]. It should be noted that the *AMPK* activity is primarily regulated through post-translational phosphorylation at Thr172 rather than transcriptional regulation [52]. Therefore, the changes in *AMPK* mRNA expression do not directly reflect its enzymatic activation status. In the present study, we evaluated *AMPK* at the transcriptional level to explore broader alterations in the hepatic lipid-metabolism gene network. Nevertheless, future studies including protein-level analyses, such as Western blot detection of phosphorylated AMPK (p-AMPK), would provide a more direct assessment of the AMPK activation and further clarify the mechanistic role of spirulina in modulating AMPK signaling.

To further characterize the metabolic phenotype, serum lipid parameters have been assessed. HFD feeding induced significant alterations in lipid markers compared with the sham controls. The total cholesterol (TC), LDL-C, and the LDL/HDL ratio were significantly higher in HFD and HFD + IR compared to sham. The spirulina pretreatment showed a trend toward an improved lipid profile in the HFD + IR condition, where TC was significantly less prominent. Mechanistically, spirulina’s hypocholesterolemic effects could be attributed, at least in part, to C-phycocyanin, which exhibits bile acid-binding capacity, reduces micellar cholesterol solubility and intestinal cholesterol uptake in vitro, and increases fecal excretion of cholesterol and bile acids in vivo [53]. Triglyceride (TG) levels, however, were not significantly different between any of the experimental and sham groups. This parameter evaluates systemic lipid metabolic alterations induced by the high-fat diet. Still, assessing liver triglyceride levels could be of higher interest, allowing a precise measurement of hepatic lipid content beyond histological observation and gene expression analysis of metabolic regulators.

Steatotic livers are particularly susceptible to IR-driven oxidative injury [54]. This is partly due to mitochondrial dysfunction and excessive ROS generation, which favor lipid peroxidation and compromise endogenous antioxidant defenses [9,53]. Within this context, increased lipid peroxidation (MDA) was reported in the current study following IR, which was less prominent than that of the HFD + IR condition. This was accompanied by the depletion of key antioxidant defenses, including reduced SOD activity and lower hepatic glutathione levels, as well as decreased *GPx* gene expression. Our results are lined up with previous studies. For instance, Yang et al. (2024) found significantly reduced SOD activity alongside elevated levels of MDA in HFD-fed mice [29]. Moreover, Tian et al. (2023) reported significantly lower hepatic GSH concentrations and significantly higher MDA levels in livers subjected to IR [55]. Furthermore, Junzhe et al. (2024) also demonstrated a marked decrease in *GPx* activity in the HFD-IR group [10]. HO-1 is a multifunctional microsomal enzyme involved in heme metabolism and is recognized for its multiple protective effects, including anti-inflammatory, anti-oxidative stress, and anti-apoptosis properties [56]. In the IR group, a stress-inducible response was detected, with increased *HO-1* expression compared with the sham group. Those results are thus in line with the established activation of *HO-1* as an adaptive cytoprotective mechanism during hepatic IR. Notably, IR-mediated induction of *HO-1* was attenuated in HFD + IR (lower than IR alone), while remaining above the sham levels, and no additional change was detected in the HFD + IR group compared with the HFD group alone. These results suggest that steatosis may attenuate the amplitude of the *HO-1* stress response during IR. Importantly, spirulina pretreatment increased the *HO-1* expression compared with both the HFD and HFD + IR groups, alongside a partial reduction in MDA and restoration of antioxidant defenses (SOD, GSH, *GPx*), supporting a broader attenuation of oxidative stress.

Hepatic IR triggers a robust sterile inflammatory response, in which damage-associated molecular patterns (DAMPs) activate innate immune signaling, notably through *TLR4* [57]. Once activated, *TLR4* in turn initiates downstream signaling cascades involving NF-κB, a master regulator of pro-inflammatory gene expression such as *IL-1β*, *TNF-α*, and *NLRP3* inflammasome [58,59].

In the current work, the immunofluorescence assay and RT-PCR-confirmed increased levels of tumor necrosis factor (TNF-α) and interleukin (IL-1β) in both the IR and HFD groups, as well as in their combination (HFD + IR). This increase was accompanied by the upregulation of Toll-like receptor 4 (*TLR4*) and nuclear factor kappa B (*NF-κB*) gene expression. In our study, the hepatic *TLR4* and *NF-κB* mRNA levels were significantly increased in the IR group versus the sham group. The highest expression was observed in the combined HFD + IR condition. TNF-α and IL-1β transcripts followed the same pattern. These findings indicate that steatosis amplifies inflammatory signaling during reperfusion [10].

In accordance with the current study’ results, clinical meta-analyses report elevated circulating pro-inflammatory cytokines, particularly TNF-α and IL-6, in patients with NAFLD compared to controls [60]. Moreover, Junzhe et al. (2024) found that the HFD-IR injury group exhibited elevated the hepatic mRNA expression of *TNF-α* and *IL-1β*, as well as significantly higher circulating levels of these cytokines, compared to the HFD-sham group [10]. Together, these results support the concept that steatosis amplifies pro-inflammatory response compared with standard diet-fed rat, which likely contributes to increased vulnerability of fatty livers to injury.

Spirulina preconditioning was associated with a significant downregulation in key hepatic inflammatory markers (*TLR4*, *NF-κB*, *TNF-α*, and *IL-1β*), supporting an overall attenuation of the hepatic inflammatory response in steatotic livers exposed to IR. Spirulina contains several bioactive constituents, including phycocyanin, heptadecane, and heptadecane. These compounds have been shown to suppresses the expression of pro-inflammatory genes by reducing *NF-κB* activity [13,61], suggesting a possible contribution, although this mechanism was not directly assessed in our model. An earlier study reported that spirulina reduces pro-inflammatory responses induced by a high-fructose diet, an effect attributed to the modulation of TNF-α and IL-6 levels [4].

Beyond the *TLR4*/*NF-κB* axis, inflammasome signaling is increasingly recognized as a key driver of sterile inflammation during hepatic IR. Pyroptotic signaling has emerged as a key amplifier of sterile inflammation in hepatic IR injury, especially in steatotic livers [10]. This process is initiated by inflammasome assembly [62] leading to caspase-1 activation, the maturation of IL-1β and IL-18, and cleavage of gasdermin D (GSDMD), which forms membrane pores [59]. In steatotic IR injury models, pyroptosis has been reported to occur mainly in macrophages [10]. Consistent with this cell-type specificity, blocking *GSDMD* processing in innate immune cells may attenuate liver IR injury [63,64]. In line with these reports, the current study’s data show that HFD + IR increased *NLRP3*, *IL-18*, and *GSDMD* mRNA levels, whereas spirulina significantly reduced these transcripts and shifted them toward sham values. Because caspase-1 activation or *GSDMD* cleavage was not assessed in the present study, these data support the modulation of the inflammasome/pyroptotic pathway at the transcriptional level.

Several limitations should be considered. Inflammasome/pyroptosis-related outcomes were limited to the mRNA levels (*NLRP3*, *IL-18*, *GSDMD*), without the assessment of caspase-1 activation, *GSDMD* cleavage, or mature cytokines. Finally, only one spirulina dose and preconditioning regimen were tested, and the contribution of specific bioactive constituents was not directly examined.

## 5. Conclusions

Overall, in this rat model of diet-induced hepatic steatosis subjected to partial hepatic ischemia–reperfusion, spirulina preconditioning attenuated liver injury. The protective effect was associated with reduced transaminase release, improved histological appearance, lower oxidative stress, downregulation of *TLR4*/*NF-κB*-related inflammatory markers with concordant protein findings for TNF-α and IL-1β, reduced expression of inflammasome/pyroptosis-related genes (*NLRP3*, *IL-18*, and *GSDMD*), and a coordinated shift in lipid metabolism-related gene expression (*SREBP-1c* and *AMPK*). Together, these findings suggest that spirulina may represent a promising nutritional approach to improving tolerance for steatotic livers to IR injury, although further mechanistic and translational studies are required.

## Figures and Tables

**Table 1:** The primary and secondary antibodies used in this study.

Antibodies	Supplier	Catalog Number	Source	Dilution
TNF alpha antibody (52B83)	Santa Cruz Biotechnology, Santa Cruz, CA, USA	sc-52746	Mouse monoclonal	1:100
IL-1β antibody (E7-2-hIL1β)	Santa Cruz Biotechnology, CA, USA	sc-32294	mouse monoclonal	1:100
Goat anti-mouse IgG (H + L) Alexa Fluor TM 488	Thermo Fisher Scientific, Emeryville, CA, USA	A-11001	Goat	1:100

**Table 2:** Gene primers used for RT-PCR.

Gene	Gene Accession Number	Forward Sequence	Reverse Sequence
*GADPH*	NM_017008.4	5′-CCTCTCTTGCTGAGTGTCCT-3′	5′-AAGAGCAACTGGGACTCTTC-3′
*NF-κB*	NM_199267	5′-ATCTTGAGCTCGGCAGTGTT-3′	5′-TCTGCTTCCAGGTGACAGTG-3′
*IL-1B*	NM_031512	5′-GAAATGCCACCTTTTGACAGTG-3′	5′-CTGGATGCTCTCATCAGGACA-3′
*TNF-A*	NM_012675	5′-CTCTTCTCATTCCCGCTCGT-3′	5′-GGGAGCCCATTTGGGAACTT-3′
*NLRP3*	NM_001191642.1	5′-TCTGTTCATTGGCTGCGGAT-3′	5′-TAGCCGCAAAGAACTCCTGG-3′
*GSDMD*	NM_001106983.1	5′-CCAGCATGGAAGCCTTAGAG-3′	5′-CAGAGTCGAGCACCAGACAC-3′
*IL-18*	NM_019165.2	5′-CAGAAGCTGGGGTTGGTGAA-3′	5′-CCCATGTCTCCAAGGGCATT-3′
*AMPK*	NM_023991.1	5′-AGTCCACGGCAGACAGAATC-3′	5′-GAAGATCGGACACTACGTGC-3′
*SREBP-1*	NM_001276707.1	5′-AGGAGGCCATCTTGTTGCTT-3′	5′-GTTTTGACCCTTAGGGCAGC-3′
*TLR4*	NM_019178.2	5′-CCAGAGCCGTTGGTGTATCT-3′	5′-CAGAGCATTGTCCTCCCACT-3′
*GPx*	NM_030826.4	5′-AACACCGTCTGGGACCTACC-3′	5′-CTCACCCGCTCTTTACCTT-3′
*HO-1*	NM_012580.2	5′-CAGAAGGGTCAGGTGTCCAG-3′	5′-GAAGGCCATGTCCTGCTCA-3′
*β-actin*	NM_031144.3	5′-TACTCCTGCTTCCTGATCCACAT-3′	5′-TATGCCAACACAGTGCTGTCTGG-3′

## Data Availability

The original contributions presented in this study are included in the article/Supplementary Materials. Further inquiries can be directed to the corresponding authors.

## Supplementary Materials

The following supporting information can be downloaded at: https://www.mdpi.com/article/10.3390/antiox15030390/s1, Figure S1: Transmitted-light photomicrographs of: negative control in liver sections, performed using preabsorbed antisera and omitting the primary antibodies; the white scale bar represents 20 μm; Table S1: Composition of rats administered food: standard diet and high-fat-diet (HFD). Basic food contains corn, soya, vitamins and minerals; Table S2: Kruskal–Wallis test for hepatic steatosis scores; Table S3: Pairwise Dunn–Bonferroni comparisons of hepatic steatosis scores; Table S4: Kruskal–Wallis test for Suzuki histological scores; Table S5: Pairwise Dunn–Bonferroni comparisons of Suzuki histological scores.
