# Supplementary material for: Spirulina Preconditioning Attenuates Ischemia–Reperfusion Injury in a Steatotic Rat Liver Model
Source: Antioxidants (Basel). 2026 Mar 19;15(3):390. doi: 10.3390/antiox15030390 (PMC13024548; doi:10.3390/antiox15030390)
Supplement: Supplementary file 1 [file antioxidants-15-00390-s001.zip › antioxidants-4193770-supplementary.pdf]

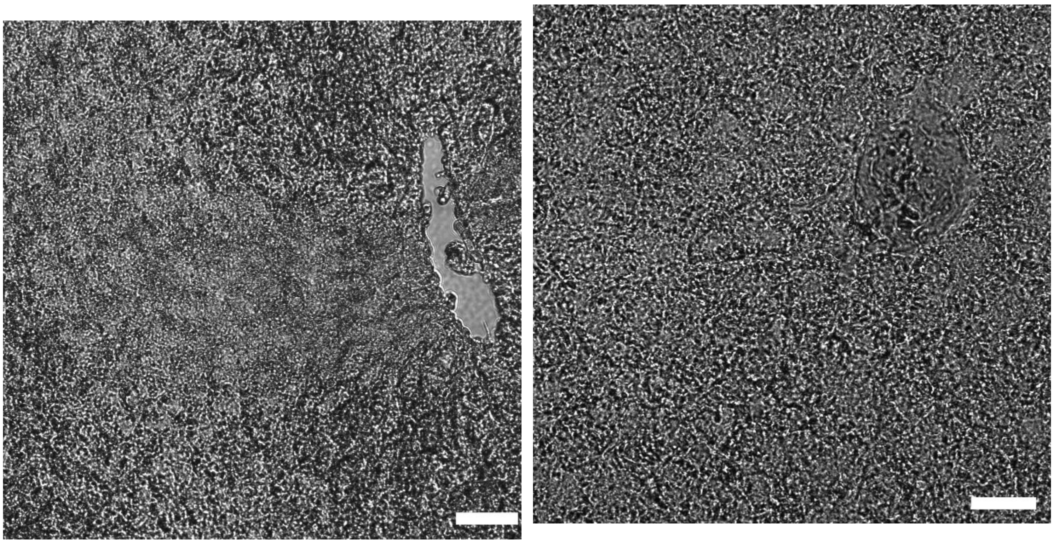

Figure S1. Transmitted-light photomicrographs of: negative control in liver sections, performed using preabsorbed antisera and omitting the primary antibodies; the white scale bar represents 20 µm.

Table S1: Composition of rats administered food: standard diet and high-fat-diet (HFD). Basic food contains corn, soya, vitamins and minerals.

		Standard diet		HFD	
		Nutritional properties (%)	Caloric value (kcal/kg)	Nutritional properties (%)	Caloric value (kcal/kg)
**Moisture**		12		8.6	
**Fibers**		3		2.155	
**Ash**		5.5		3.944	
**Lipids**		4	360	31.17	2805
**Proteins**		19.5	780	13.98	559
**Carbohydrate**		55	2200	39.44	1577
**Total**			3340		4941
Total energy intake/rat/day					
SD			HFD		
≈ 59.4 kcal			≈ 98.8 kcal		
**Amino acids (%)**					
	Methionine	0.44		0.44	
	Cysteine	0.35		0.35	
	Threonine	0.78		0.78	
	Tryptophane	0.25		0.25	
**Mineral mix (mg/kg)**					
	Manganese	70		70	
	Iron	40		40	
	Copper	20		20	
	Zinc	52		52	
	Selenium	0.24		0.24	
	Cobalt	0.16		0.16	
	Iode	0.96		0.96	

**Vitamins and Antioxidants (per kg diet)**			
	Vitamin D3	2800 IU	2800 IU
	Vitamin A	10400 IU	10400 IU
	Vitamin H	32 mg	32 mg
	Antioxidants (BHA-BHT)	125	125

BHA: Butylated hydroxyanisole; BHT: Butylated hydroxytoluene

Table S2: Kruskal–Wallis test for hepatic steatosis scores.

Variable	Test	χ^2	df	p-value
**Steatosis score**	Kruskal–Wallis	27.964	4	<0.001

Table S3: Pairwise Dunn–Bonferroni comparisons of hepatic steatosis scores

Comparison	Z statistic	Adjusted p-value	Significance
**Sham vs IR**	0.000	1.000	ns
**Sham vs SP1000**	0.000	1.000	ns
**Sham vs HFD**	-3.413	0.006	*
**Sham vs HFD+IR**	-3.413	0.006	*
**IR vs SP1000**	0.000	1.000	ns
**IR vs HFD**	-3.413	0.006	*
**IR vs HFD+IR**	-3.413	0.006	*
**SP1000 vs HFD**	3.413	0.006	*
**SP1000 vs HFD+IR**	3.413	0.006	*
**HFD vs HFD+IR**	0.000	1.000	ns

* Significant after Bonferroni correction ($p < 0.05$)

Table S4: Kruskal–Wallis test for Suzuki histological scores.

Variable	Test	χ^2	df	p-value
**Steatosis score**	Kruskal–Wallis	18,941	3	<0.001

Table S5: Pairwise Dunn–Bonferroni comparisons of Suzuki histological scores.

Comparison	Z statistic	Adjusted p-value	Significance
**Sham vs IR**	-2.372	0.177	ns
**Sham vs SP1000**	-1.326	1.000	ns
**Sham vs HFD**	-3.524	0.004	*
**Sham vs HFD+IR**	-4.291	0.000	*
**IR vs SP1000**	1.047	1.000	ns
**IR vs HFD**	-1.151	1.000	ns
**IR vs HFD+IR**	-1.919	0.550	ns
**SP1000 vs HFD**	2.198	0.280	ns
**SP1000 vs HFD+IR**	2.965	0.030	*
**HFD vs HFD+IR**	-0.768	1.000	ns

* Significant after Bonferroni correction ($p < 0.05$)
